# Microbial fuel cells: From fundamentals to applications. A review

**DOI:** 10.1016/j.jpowsour.2017.03.109

**Published:** 2017-07-15

**Authors:** Carlo Santoro, Catia Arbizzani, Benjamin Erable, Ioannis Ieropoulos

**Affiliations:** a Department of Chemical and Biological Engineering, Center Micro-Engineered Materials (CMEM), University of New Mexico, 87106, Albuquerque, NM, USA; b Department of Chemistry “Giacomo Ciamician”, University of Bologna, Via Selmi 2, 40126, Bologna, Italy; c University of Toulouse, CNRS, Laboratoire de Génie Chimique, CAMPUS INP – ENSIACET, 4 Allée Emile Monso, CS 84234, 31432, Toulouse Cedex 4, France; d Bristol BioEnergy Centre, Bristol Robotics Laboratory, T Block, University of the West of England, Frenchay Campus, Coldharbour Ln, Bristol, BS16 1QY, United Kingdom

**Keywords:** Microbial fuel cell, Electroactive biofilm, Microbial fuel cell anode, Microbial fuel cell cathode, Cathode reaction mechanisms, Microbial fuel cell practical applications

## Abstract

In the past 10–15 years, the microbial fuel cell (MFC) technology has captured the attention of the scientific community for the possibility of transforming organic waste directly into electricity through microbially catalyzed anodic, and microbial/enzymatic/abiotic cathodic electrochemical reactions. In this review, several aspects of the technology are considered. Firstly, a brief history of abiotic to biological fuel cells and subsequently, microbial fuel cells is presented. Secondly, the development of the concept of microbial fuel cell into a wider range of derivative technologies, called bioelectrochemical systems, is described introducing briefly microbial electrolysis cells, microbial desalination cells and microbial electrosynthesis cells. The focus is then shifted to electroactive biofilms and electron transfer mechanisms involved with solid electrodes. Carbonaceous and metallic anode materials are then introduced, followed by an explanation of the electro catalysis of the oxygen reduction reaction and its behavior in neutral media, from recent studies. Cathode catalysts based on carbonaceous, platinum-group metal and platinum-group-metal-free materials are presented, along with membrane materials with a view to future directions. Finally, microbial fuel cell practical implementation, through the utilization of energy output for practical applications, is described.

## Highlights

• The history of MFCs in the context of bioelectrochemical system is introduced.
• Electroactive biofilms and electron transfer mechanisms are described.
• Carbonaceous and metallic anode materials are presented.
• Cathode catalysts are presented and ORR mechanisms are described.
• Utilization of MFC energy output for practical applications is described.

## Graphical abstract

**Figure:**
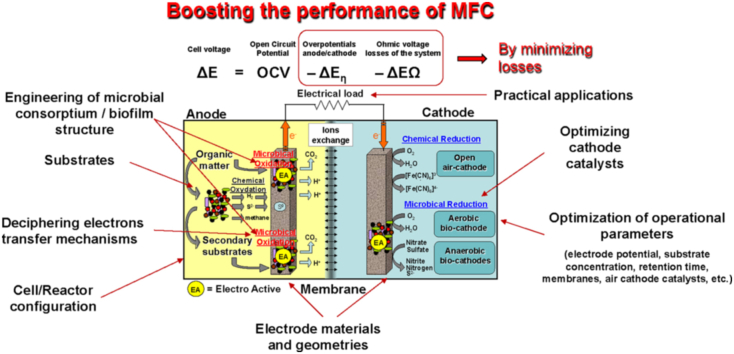

## 1 Introduction

**Fig. 1:**
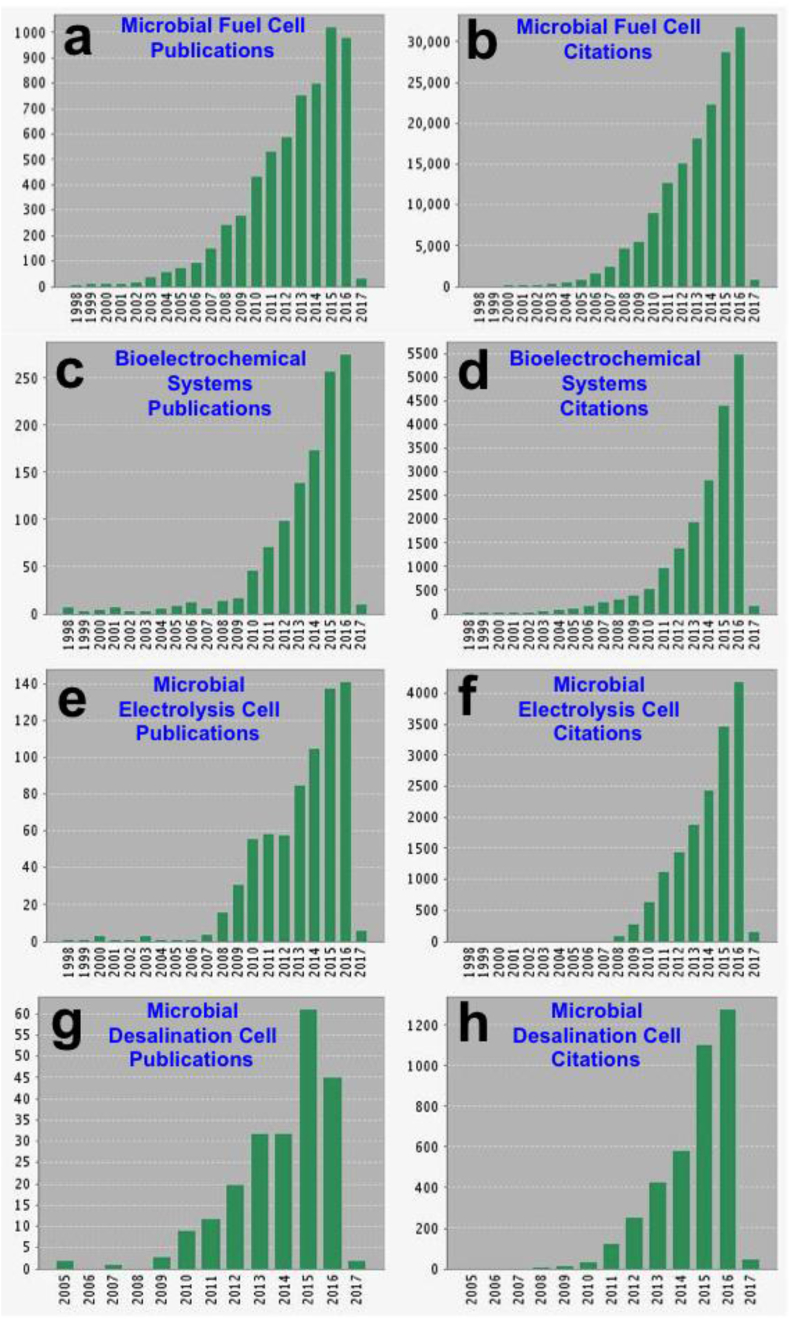
Quantitative analysis of the scientific literature on microbial fuel cells and bioelectrochemical systems (Source: ISI WEB OF SCIENCE, January 2017).

Microbial Fuel Cells (MFCs) (Fig. 1a and b) and more recently extended into various Bio-Electrochemical Systems (BESs) (Fig. 1c and d) are an interesting and constantly expanding field of science and technology that combines biological catalytic redox activity with classic abiotic electrochemical reactions and physics [1], [2], [3], [4].

The addition of biological organisms responsible for catalyzing electrochemical reactions, gives these systems a level of complexity that is perhaps above that of already complex electrochemical systems (e.g. batteries, fuel cells and supercapacitors). The main differences of MFCs with the conventional low temperature fuel cells (direct methanol fuel cell or proton exchange membrane fuel cell) are: i) the electrocatalyst is biotic (electroactive bacteria or proteins) at the anode [5], [6], [7]; ii) the temperature can range between 15 °C and 45 °C, with close to ambient levels as optimum [8], [9], [10]; iii) neutral pH working conditions [11], [12], [13], [14]; iv) utilization of complex biomass (often different types of waste or effluent) as anodic fuel [15], [16]; v) a promising moderate environmental impact assessed through life cycle analysis [17], [18].

The original idea of utilizing microbes to generate electricity was conceived and attributed to Potter in 1911 [19], even though the concept of ‘animal electricity’ dates back to the 18th century, when Galvani was experimenting with frog legs [20]. Further concepts and practical developments were explored since, with Cohen's 35-unit setup in 1931 [21], Karube et al. catalyst investigations in the 60's [22] and more recently in the 80s-90s, with the work of Bennetto et al. on synthetic mediators, which resulted in the development of the so-called “analytical MFC” that is still in use to date [23]. From those early examples, significant progress on the understanding of electron transfer mechanisms, development of efficient bio-electrocatalytic interfaces and development of novel, low cost and durable electrode materials, has already been achieved but there is nevertheless ample room for improvement and work to be done, before reaching industrialization of MFCs [24], [25].

Several BESs have been proposed and they are classified by applications (Fig. 1). The first one and the most studied, is MFC representing over 75% publications in 2016 according to the ISI OF WEB OF SCIENCE (Fig. 1a). This type of BES can extract chemical energy from complex organic substrates and convert it into useful electricity [1], [26]. Other BESs have been developed that generate useful products (e.g. hydrogen [27], [28], [29], formate [30], [31], acetate [32], [33], methane [34], [35], [36], etc [37], [38]) or desalinate water (e.g. microbial desalination cell [39], etc [40], [41], [42]).

Numerous challenges still remain unsolved in the MFC field for successful deployment in real environments, although some initiatives have been reported [43]. Starting from the anode, remarkable progress has been made with synthetic substrates and model microbial catalysts or microbial consortia developed in the laboratory. However research questions of implementation are inherently more complicated when it comes to working in more complex conditions such as real industrial effluents [15], [16] or natural environments (sediments [44], [45], [46], [47], [48], marine environments [49], [50], [51], lagoons [52], [53], [54], [55], etc.). As an example, several types of organic waste have been used as fuel for microbial anodes [15], [16], but electroactive bacteria kinetics remains poor and the interaction between electrode and bacteria has still not been fully understood [56], [57], [58]. Moreover, interaction and/or coexistence in electron transfer mechanisms between bacteria and solid electrodes are not well described, especially in complex environments in which a multitude of microbial species (electroactive or not) can be found on the electrodes [59], [60], [61], [62], [63]. Finally, the attraction of microbial cells towards the electrodes [64], biofilm formation and development on anode surface [64], [65], interaction and inter-species cooperation [60], [61], [62], [63], [66], [67], [68], [69], as well as influence of environmental parameters on microbial colonization [4], [25], remain unknown due to the difficulty of coupling the complicated processes of microbial electrochemistry and the existing imaging technology [70], [71], [72], [73], [74]. To a certain extent, the interaction of bacteria with electrode surfaces has been studied by varying the surface morphology and chemistry [75].

At the cathode, oxygen has primarily been used as the oxidant due to its abundance and high reduction potential [76], [77]. Some studies showed also the possibility of utilizing metallic oxidants (e.g. U [78], [79], Cd [80], Cr [81], [82], Cu [83], [84] etc.) that can be reduced to a less toxic oxidation state. The oxygen reduction reaction (ORR) remains one of the main bottlenecks of this technology, due to the high over-potentials and low kinetics that are encountered [76].

A further challenge is related with the low energy produced by MFCs, which is currently orders of magnitude lower compared to that of chemical fuel cells. The harvesting and management of the low power generated by MFCs has given rise to new hybrid systems that partially address this problem by coupling MFCs with external off-the-shelf harvesting systems based mainly on supercapacitors [85], with a number of applications reported (See Section 2.8). Recently, capacitive features of the electrodes have been investigated [86], [87], [88] and supercapacitive electrodes have also been used as internal supercapacitors and the properties of those materials have been studied [89], [90], [91], [92], [93].

Finally, several organic compounds coming from different municipal and industrial types of wastewater have been successfully investigated showing the feasibility of BES in generating power and simultaneously degrading pollutants, thus becoming an alternative technology for cleaning water with zero or positive energy budget [15], [16].

In this review, the authors describe briefly the important steps that moved the electrochemical abiotic field towards the biological-electrochemical hybrid. The authors wish to communicate the level of progress achieved in: i) evolution of bioelectrochemical systems from MFC to MXC; ii) understanding of the microbiology of anode and the electron transfer mechanisms in electroactive microbial biofilm, iii) electrochemistry regarding the anode and cathode, iv) electrode materials research and development and v) practical applications involving MFCs.

## 2 Discussion

### 2.1 From abiotic fuel cell to biological and microbial fuel cell

Luigi Galvani, physician and professor at the University of Bologna, is historically considered to be the first electrochemist and bioelectricity pioneer [20]. In fact with his experiments in 1780, he discovered that the muscles of dead frog legs moved (or twitched) when struck by an electrical spark and coined the term “animal electricity” to describe the force that activated the muscles of his specimens as being generated by an electrical fluid that is carried to the muscles by the nerves. Alessandro Volta, a contemporary professor of experimental physics at the University of Pavia, checked Galvani's experiments and believed that the contractions occurred due to the metal cable Galvani used to connect nerves and muscles in his experiments. The Galvani-Volta controversy grew fervent at the end of the 18th century and was the platform that led shortly to the invention of an early battery, resulting from Volta's experiments [94]. Significant advancements dealing with electrochemical systems for power generation or energy storage have been carried out in several areas during the first few decades of the 19th century. It is important to cite, among the breakthroughs in electrochemical devices, the lead-acid battery that was invented in 1859 by the French physicist Gaston Planté, which is still playing a key role in the battery market, and the first H_2_/O_2_ acid fuel cell by the Welsh lawyer turned scientist William Grove. Grove is recognized to be the father of fuel cells: in fact, in a letter published in 1838 on *The London and Edinburgh Philosophical Magazine and Journal of Science* he wrote about the development of his “gas battery” [95] that inspired several scientists. Although in *Electrochemistry: History and Theory,* published in 1896, Wilhelm Ostwald described Grove's gas battery as “of no practical importance but quite significant for its theoretical interest”, in 1889 Charles Langer and Ludwig Mond coined the term “fuel cell” as they were trying to engineer the first practical fuel cell using air and coal gas. In 1932, a century after Grove's experiments, Francis Bacon developed the first successful hydrogen-oxygen fuel cell with alkaline electrolyte and in 1959 demonstrated a practical 5 kW system [96]. Advancements in fuel cells (FCs) were achieved in subsequent years, also with the involvement of NASA as well as of national agencies and vehicle manufacturers. A variety of fuel cells were developed and usually classified by function of the electrolyte utilized (polymeric membrane, ceramics, liquid electrolyte). Often they are identified according to the fuel type, as is the case of alcohol fuel cells, and also according to the operating pH level (alkaline or acidic). The electrolyte and the electrode used determine the operating temperature (60–200 °C (low temperature) and 600–1000 °C (high temperature)) [96]. All types of fuel cells have advantages and disadvantages. The high operating temperature of solid oxide fuel cells (SOFCs) and molten carbonate fuel cells (MCFCs) is a limit for the slow start-up times but provides an advantage in removing the need for precious metal catalysts, thereby reducing cost. Additionally, waste heat from SOFCs and MCFCs may be captured and reused, increasing the theoretical overall efficiency up to 85%. The low-temperature proton exchange membrane fuel cells (PEMFCs) usually work at temperatures within the 60–110 °C range. This relatively low temperature is sufficient to improve the kinetic processes without degradation phenomena occurring. However, precious metals are needed as catalysts to enhance the H_2_ dissociation rate at the anode and, to a greater extent, to accelerate the decomposition of the stable intermediate H_2_O_2_ that is produced at the cathode during the two-step O_2_ reduction reaction. The replacement of precious metal catalysts is still a challenge to date [97], [98].

The advantages of these FCs are usually masked by one or more of the aforementioned challenges, i.e. high temperatures, high cost and in some cases, highly corrosive media. In this regard, BFCs are attractive since they operate under mild reaction conditions, namely ambient operational temperature and pressure, employ neutral or circumneutral electrolytes and use inexpensive catalysts and anodic fuel that can range from simple organic molecules like glucose or acetate to complex organic waste, like waste waters and urine [99], [100], [101], [102].

BFCs could be defined as devices able to transform chemical to electrical energy via electrochemical reactions involving biochemical pathways and can be divided into enzymatic fuel cells (EFCs) [103], [104], [105] and MFCs [1]. The former use selective enzymes to perform redox reactions that produce current while the latter utilize electroactive microbes to degrade organics and produce electricity. Generally, enzymes have better electrochemical catalytic performance but are unsustainable and less durable compared to microbes. The first report of an actual MFC dates back to the beginning of last century, when the English botanist Michael Cresse Potter demonstrated that microorganisms could generate a voltage and deliver current [19]. Biological fuel cells became popular in the 1960s, when NASA showed short-term interest in turning organic waste into electricity on space missions. Interest in BFCs was then reinvigorated in the 80′ following Bennetto et al., who put emphasis on the MFC functionality with a focus on mediator-based electron transfer [106], [107]. Since the beginning of the 21st century, interest in MFCs has been growing exponentially, as illustrated by the number of publications and related citations (see Fig. 1).

BFCs convert the chemical energy of organics directly into electrical energy and use either a microorganism or an enzyme as the catalyst [108], [109], [110], [111]. Enzymes possess remarkable advantages over chemical catalysts, such as biocompatibility, higher transformation efficiency, higher activity under mild conditions and particularly higher specific selectivity. The last two features enable the BFC to operate without a separation membrane, a factor that makes miniaturization possible and, in turn, the prospect of using enzymatic fuel cells in wearable and implantable devices, feasible [109], [112], [113]. Unfortunately the enzymatic life time is short and it is even further shortened in the presence of pollutants [109], [114], [115]. The development of mediatorless enzyme-based biocathodes and bioanodes has addressed one of the issues of EFC, i.e. the use of mediators. Also the increase in the active lifetime of the immobilized enzymes through encapsulation in micellar polymers that avoid enzyme denaturation and provide a biocompatible hydrophobic and pH-buffered environment contributed to the development of EFC [105], [116], [117], [118]. The use of whole microbial cells in MFC for the bioelectrochemical oxidation of fuels is advantageous since it eliminates the need for enzyme isolation and still allows multiple enzymatic reactions to take place in conditions close to their natural environment, with the organisms regenerating the required enzymes as part of their natural life. On the other hand, they have a slower response time owing to the more complex chemical pathways. Although MFC target applications could span across scales, in general they differ from those for EFCs: MFCs can be typically envisaged for large-scale applications for wastewater treatment [119], [120] or in small-scale for small and portable applications [121], EFCs are instead compact, miniaturized and flexible bioelectrochemical devices [105], [122].

### 2.2 From microbial fuel cells to bioelectrochemical systems

**Fig. 2:**
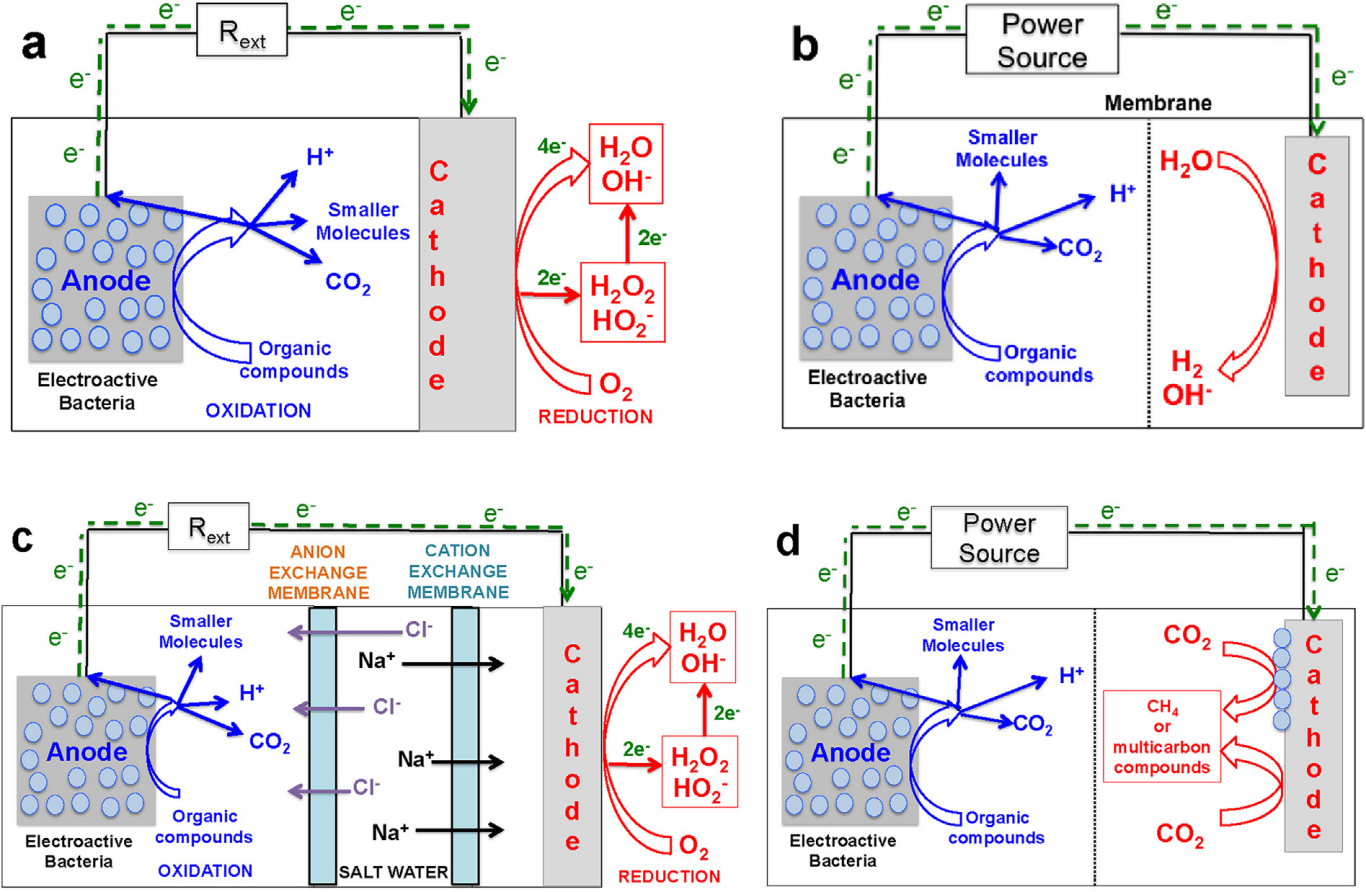
Schematic of a microbial fuel cell (a), microbial electrolysis cell (b), microbial desalination cell (c) and general microbial electrosynthesis cell (d).

As mentioned before, MFCs are by far the most studied and reported BESs (Fig. 1a and b). The main motive for pursuing this technology is the potential for complementing the existing costly wastewater treatment systems with a technology that can actually be self-sustainable or even have a net positive energy output while pollutants are removed. A general schematic diagram of the microbial fuel cell is presented in Fig. 2 a. In parallel, several other bioelectrochemical systems of interest have been developed (Fig. 2b–d) [79], [123], [124], [125], [126].

Among them, one of the most interesting and well investigated, is the Microbial Electrolysis Cell (MEC), which was firstly introduced in 2005 [127]. Related publications on MEC increased over time to 141 in 2016 (Fig. 1e) with over 4000 citations (Fig. 1f). A depiction of the microbial electrolysis cell is here presented (Fig. 2b). MEC requires an external source of electricity for electrolysis to produce hydrogen at the cathode, but this external energy supply is of a small amount, since most of the energy comes from the chemical energy extracted from substrates oxidized at the anode [128]. Consequently, hydrogen can be produced with a low consumption of energy utilizing bioelectrocatalysis supported by additional low energy power sources. MEC is of a particular interest since hydrogen is a precious gas produced and fundamentally needed for the upcoming hydrogen-energy economy [129], [130]. Several developments and improvements concerning the increase in hydrogen production [131], [132], [133], [134], [135], improvements in cell design [136], [137], [138], removal of membrane [139], [140], [141], [142], utilization of microbial catalysts [143], [144], [145] or Pt-free catalysts [146], [147], [148], [149], [150], [151], [152], [153], [154] have been successfully demonstrated. Recently a large-scale application has also been shown, dealing with the production of hydrogen from winery wastewater [155]. Recently, three examples have dealt with relatively large scale (order of magnitude of one liter and above) MECs [156], [157], [158]. This clearly indicated the intention of scientists to scale up the MEC systems towards practical applications, through studying the limitations related to the increase in reactor size.

Most recently, other BESs have been developed with cogenerative and trigenerative purposes. Among them, interestingly, microbial desalination cell (MDC) has been successfully developed with the tentative objective of treating wastewater, generating electricity and desalinating water simultaneously [39]. A general schematic of the microbial desalination cell is here presented (Fig. 2c). The increasing interest in this particular topic is highlighted by the growing number of publications (Fig. 1g) and related citations (Fig. 1h). Recent reviews work reports on the main configurations adopted in microbial desalination cells [159], [160], [161], [162], with the implementation of air-breathing [163], [164], [165] as well as bio-cathodes [166] and osmotic membranes [167] have been used. Also other parameters were investigated for improving microbial desalination cells such as recirculating anolyte and catholyte [168], stacking the cells [169], [170] and using capacitive materials for deionization [171], [172], [173]. A pilot MDC system of 105 L was also recently presented [174].

In parallel with this research activity, BESs have recently been presented as microbial electro-synthesis devices in which specific bacteria or operating conditions allowed the production of valuable products from CO_2_ or other compounds, including gas transformation or reduction. This relatively new direction is of high interest because of the possible utilization of renewable energy when disconnected from the main power lines distribution. The main principles of the microbial electrosynthesis cell are shown in Fig. 2 d. Interestingly, CO_2_ can be transformed to methane [37], [38], [175], acetate [37], [38], formate [176] and other compounds [37], [38], [177], [178], [179], [180]. While the feasibility of the process has been shown in several cases, numerous problems have still to be overcome. Among them, selectivity of the product, separation of the product from the solution, low reaction kinetics and cell design seem to be most challenging to address. Despite these difficulties, results are quite encouraging and deserve further investigations.

### 2.3 Electroactive (EA) biofilms: the microbial electrocatalysts of bioelectrochemical systems

EA biofilms (also called electrochemically-active biofilms) have been identified in a large variety of natural ecosystems such as soils, sediments, seawater or freshwater but also in samples collected from a wide range of different microbially-rich environments (sewage sludge, activated sludge, or industrial and domestic effluents). One aspect of the electro-catalytic ability of biofilms is related to the presence of some specific bacterial strains (*Geobacter sulfurreducens*, *Rhodoferax ferrireducens*, *Shewanella* sp., etc.) that are able to exchange electrons with solid substrata (i.e. electrodes) [5], [110].

#### 2.3.1 Mechanisms of electrons transfer with solid electrodes

**Fig. 3:**
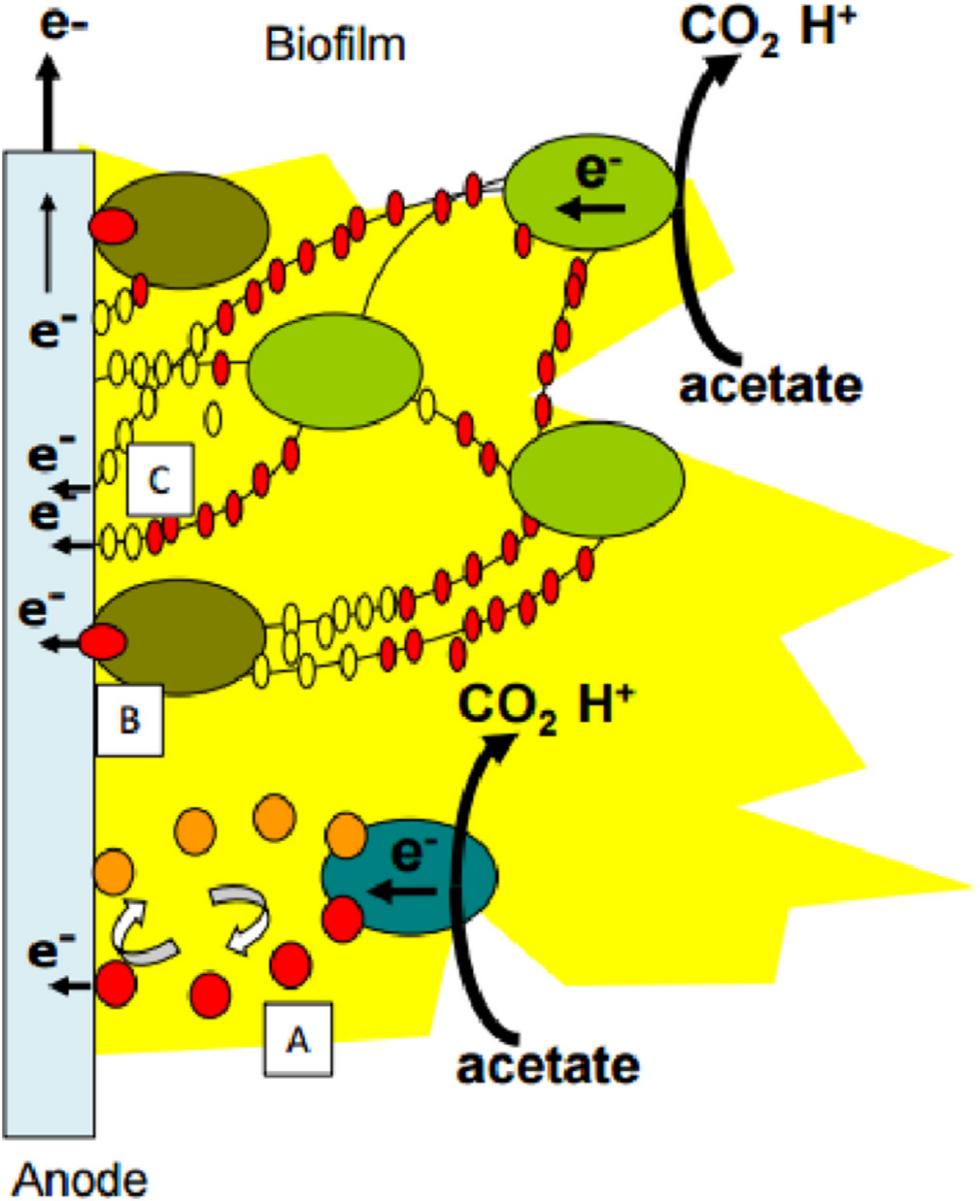
Mechanisms involved in electron transfer: (A) Indirect transfer via mediators or fermentation products; (B) direct transfer via cytochrome proteins; (C) direct transfer via conductive pili.

The transfer of electrons between EA bacteria entrapped inside the EA biofilms and an electrode can be direct or indirect [181] (Fig. 3). Many bacteria such as *Shewanella oneidensis*, *Pseudomonas alcaliphila*, *Pseudomonas aeruginosa* can produce their own redox mediators. For example pyocianine (pigment) has been identified as responsible for the electrochemical activity in *P. aeruginosa* [111]. For *S. oneidensis* the production of a quinone mediator (2-amino-3-dicarboxy-1,4 naphthoquinone) increases by a factor of 2 the power density of a MFC compared with a MFC without the mediator [182]. Indirect electron transfer may also be performed via the oxidation of a by-product resulting from bacterial metabolism. An example is the hydrogen produced by fermentative bacteria and which is then oxidized at the surface of the anode [183].

Direct electron transfer between the electroactive bacteria and the electrode occurs by direct contact between the outer membrane of the bacteria and the surface of the anode [48]. The ultimate exchange of electrons, between the cell and the electrode, is provided by the cytochrome *c* membrane proteins. The direct transfer mechanism via the cytochrome has been clearly demonstrated in *G. sulfurreducens* through experiments with mutants wherein the gene encoding the cytochrome *c* proteins has been deleted or overexpressed [184]. More recently in 2005, an additional direct transfer mechanism has been described, which takes place via extracellular conductive connections called conductive pili or bacterial nanowires [185]. In the current state of knowledge, it is impossible to separate the electron transfer mechanisms involved in mixed species biofilms. The mechanistic approach to elucidate the redox steps of electronic transfers requires working with model microorganisms (pure strains), under strictly controlled conditions, and quite possibly preceded by simulation modelling.

#### 2.3.2 Formation of EA biofilms

##### 2.3.2.1 Supply of electrolyte

EA biofilms formation has been performed in batch, fed-batch, or continuous-flow modes, fed-batch being the most common practice so far. Fed-batch is often conducted by adding a fresh dose of substrate when the electrical output falls below a baseline [186], [187], [188], or by replacing the whole solution [189] or a part of the solution [190], [191]. The repeated addition of an inoculum is in some cases necessary in the first batches, and then only fresh medium is added in further replacements/replenishments [192]. A different procedure that avoids current decrease by maintaining substrate concentration above a given threshold has sometimes led to higher performance [193], [194]. The two procedures have also been associated, with medium replacement after 24 h and then successive substrate additions that kept the current near maximum [195].

Continuous mode has been implemented with hydraulic residence times of the order of 12–20 h [196], [197], [198]. Biofilm formation starts in general with the inoculum added as part of the medium in batch mode [199], [200] or inside a recirculation loop for 24 h to a few days [201]. The continuous feeding phase is then performed by providing the reactor with only the carbon-energy medium. Continuous mode is particularly interesting because it ensures a more stable electrolyte environment and allows controlled changes in its composition.

##### 2.3.2.2 Chronoamperometry to form bioanodes

EA biofilms formation under chronoamperometry is almost universally carried out at a constant applied potential (−0.6 V to +0.5 V vs. SHE) [202]. Only rarely studies have attempted to form EA biofilms with electrode potential changes [203], [204], or with a first phase at open circuit before establishing the polarization potential [205], [206]. Bioanodes formed in arctic soils have shown similar microbial communities in the biofilms that developed at open circuit or under polarization (0.1 V vs SHE) [207] but microbial colonization was considerably lower at open circuit for bioanodes formed from marine sediments (0.14 V vs. SHE) [208] or garden compost (0.04 V vs. SHE) [206].

#### 2.3.3 Enrichment of biofilms in EA bacterial species

The strategies of EA species enrichment in biofilms are generally applicable for complex inocula and are designed to promote the specific development of EA species within the EA biofilm. There are two main enrichment strategies employed, which are described below.

##### 2.3.3.1 Acclimation of the inoculum

The purpose of the acclimation of the inoculum is to promote the growth of EA micro-organisms initially present in the inoculum. Among many, the most recognized methods of acclimation that optimize the selection of the EA species are (i) the addition of vitamins, essential nutrients, substrates, etc. [209], [210]; (ii) the absence of oxygen i.e. anaerobic conditions [210], [211]; (iii) the specific elimination of microbial groups by physical methods (ultrasound, temperature, etc.) or by chemical methods (antibiotics, fungicides, etc.) [212]; (iv) the chemical modification of the inoculum (change in conductivity, pH, etc.) [213].

##### 2.3.3.2 Transplanting successive generations of EA biofilms

Transplanting of EA biofilms is a technique of cultivation of successive generations of biofilms on a solid or porous conductive support (electrodes, metallic particles). Biofilms used may be mature communities collected from natural environments (sediments, soil, etc.) or from an already colonized electrode. This strategy allows less microbial diversity within the biofilm, by eliminating non-EA or non-attached to electrodes bacteria (planktonic bacteria).

EA biofilm transplanting technique has been studied for the first time by Rabaey et al., in 2004 [111]. The procedure consisted of several successive operations of EA biofilm transplanting: the biofilm on the anode of the MFC was scraped and used to inoculate a new MFC [111]. The power density of the second MFC had increased from 0.6 W m^−2^ to 4.3 W m^−2^ and the coulombic efficiency from 4 to 81%. Erable et al. used a biofilm collected under a floating metallic dock in a harbor [208]. This wild biofilm, tested under chronoamperometry at −0.1 V vs. SCE on a graphite electrode and with acetate as a fuel, had achieved a power density of 2.5 A m^−2^ while the inoculum collected in neighboring sediments gave current densities less than 1.0 A m^−2^.

In the same category, the Schroeder group has formed so called secondary EA biofilms [214], [215], [216] using the full primary microbial anode formed from wastewater to inoculate a fresh reactor. Secondary EA biofilm was produced in a sterilized synthetic medium, inoculated with only the primary bioanode maintained under a fixed potential [214]. This procedure led to highly reproducible results [191].

#### 2.3.4 The different substrates for EA biofilms

Heterotrophic bacteria are capable of oxidizing a wide variety of organic molecules (substrates) by producing useful energy for their growth and maintenance of their metabolism. The substrate then serves for the bacteria as a source of carbon-energy. The substrates used by EA biofilms can be any kind of organic matter from simple molecules (glucose, acetate, carbohydrates, etc.) to complex compounds (cellulose, molasses, etc.) as well as the organic matter contained in the wastewater treatment plants, agricultural wastes (dairies, manure, etc.), domestic wastes and any type of fermentable substrates. Two reviews summarize the substrates used [15], [16].

The efficiency of the bioelectrochemical conversion of the organic substrate into energy depends on composition, characteristics and concentration. The nature of the substrate(s) affects the composition of bacterial populations that grow within the EA biofilms, but also the bioelectrochemical performance, as the current density or the coulombic efficiency of bioanodes [217].

In the majority of studies relating to BES, acetate is the main substrate for the production of electrons at the bioanode [48], [145], [198], [218], [219], [220], [221]. Acetate is a simple substrate and the end product of fermentation for many metabolic pathways based on the oxidation of complex carbon sources: acetic fermentation from ethanol (vinegar production) or the Entner Doudoroff route from glucose, acidogenesis from the complex organic matter (protein, saccharides, lipids, etc.). Acetate has been reported to be a better substrate for electricity generation [217], when compared to butyrate, propionate or glucose. Glucose or lactate are two substrates conventionally used in studies involving electro-microbial systems [222], [223], [224] but also the first EcoBot, which was fed with glucose [225]. Pant et al. [15], [16] carefully detailed the list of different substrates used by anodic EA biofilms: arabitol, cysteine, ethanol, propionate, fumarate, starch, artificial wastewater, etc. However, in other more applied studies, the work is oriented to the use of real raw effluent at the bioanode, so that it can be closer to industrial applications or processes. Pant et al. [15], [16] also reported the use of real urban (sludge, organic waste) or industrial (brewery, chocolate, stationery, piggery) wastewater in their review paper.

### 2.4 Anode materials

The materials used as anode electrodes must have several specific characteristics for improving interactions between the EA biofilm and the material surface. The most important characteristics are: i) electrical conductivity; ii) resistance to corrosion; iii) high mechanical strength; iv) developed surface area; v) biocompatibility; vi) environmentally friendly and vii) low cost as identified in previous reviews [4], [75], [226].

**Fig. 4:**
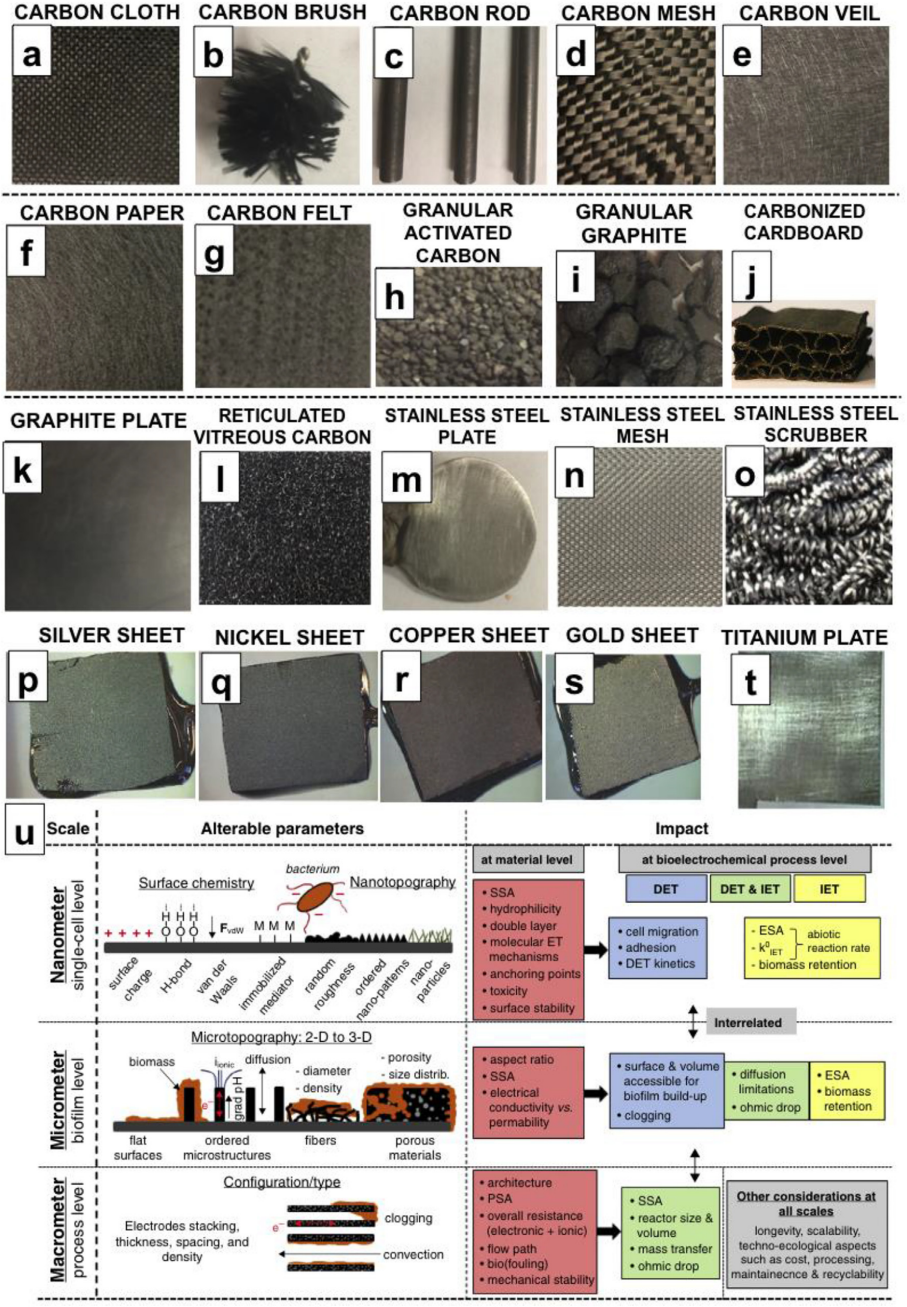
Digital photographs of carbon cloth (a), carbon brush (b), carbon rod (c), carbon mesh (d), carbon veil (e), carbon paper (f), carbon felt (g), granular activated carbon (h), granular graphite (i), carbonized cardboard (j), graphite plate (k), reticulated vitreous carbon (l), stainless steel plate (m), stainless steel mesh (n), stainless steel scrubber (o), silver sheet (p), nickel sheet (q), copper sheet (r), gold sheet (s), titanium plate (t). Effect of the chemistry and morphology on the surface characteristics and bioelectrocatalysis (u) (Fig. 4j adapted from Ref. [228], published by Frontiers, CC BY 3.0 (https://creativecommons.org/licenses/by/3.0/); Fig. 4p, q, r, s adapted from Ref. [229], published by The Royal Society of Chemistry, CC BY 3.0 (https://creativecommons.org/licenses/by/3.0/); Fig. 4t adapted from Ref. [230] with permission of Elsevier; Fig. 4u adapted from Ref. [75] with permission of Elsevier).

Carbonaceous- and metallic-based materials are the main types of electrode adopted, which possess all the above-mentioned characteristics [202], [227]. Among carbonaceous materials, carbon cloth (Fig. 4a), carbon brush (Fig. 4b), carbon rod (Fig. 4c), carbon mesh (Fig. 4d), carbon veil (Fig. 4e), carbon paper (Fig. 4f), carbon felt (Fig. 4g), granular activated carbon (Fig. 4h), granular graphite (Fig. 4i), carbonized cardboard (Fig. 4j) [228], graphite plate (Fig. 4k) and reticulated vitreous carbon (Fig. 4 l) are used as commercially available anode electrode material. Among metals-based materials, stainless steel plate (Fig. 4 m), stainless steel mesh (Fig. 4 n), stainless steel scrubber (Fig. 4 o), silver sheet (Fig. 4 p) [229], nickel sheet (Fig. 4 q) [229], copper sheet (Fig. 4 r) [229], gold sheet (Fig. 4 s) [229] and titanium plate (Fig. 4b) [230] were used as commercially available anode electrode material.

Carbon cloth is a carbonaceous material used very often as anode material in MFCs [231], [232], [233], [234], [235]. This material guarantees high surface area and relatively high porosity demonstrating also high electrical conductivity, as well as flexibility and mechanical strength, in forming more complex 3D structures. The negative aspect is related to the cost that is generally quite high.

Carbon brush is a very interesting material based on a titanium core in which carbon fibers are twisted [236], [237], [238], [239], [240]. The surface area is quite high with an optimal area to volume ratio. The high electrical conductivity is guaranteed by the central titanium metal that at the same time increases the material cost. Carbon brushes are heavily used as anodes and ongoing investigations are looking to bring down the overall costs [241], [242].

Carbon rods are mainly used as current collectors not as anode electrodes as such, due to their low surface area [243], [244]. Their cost is quite affordable for MFCs applications.

Carbon mesh is also a carbonaceous material that is commercially available, relatively low-cost and with a relatively low electrical conductivity [245], [246]. The main problem is related with the low mechanical strength that could lead to low durability under high flow conditions. Carbon mesh can be also folded to make a 3-D electrode, but its porosity is low.

Carbon veil is a very cheap carbonaceous material with relatively high electrical conductivity and high porosity [214], [247], [248], [249], [250], [251]. The latter is of extreme importance for allowing bacteria to access and colonize all the available material sites. The single layer of carbon veil is quite fragile but since the material is versatile, it can be folded to form a robust and porous 3-D electrode as shown in the literature [252], [253], [254].

Carbon paper is a planar carbonaceous material, relatively porous but expensive and fragile with mainly lab scale demonstrations under batch conditions [255], [256].

Carbon felt is a carbonaceous material that is commonly used as anode in MFC. Its characteristics are high porosity and high electrical conductivity. Similar to carbon veil, the large pores allow bacteria to penetrate through the structure and colonize the biofilm also internally. The cost is relatively low and the mechanical strength is high depending on the thickness of the material [257], [258], [259], [260], [261].

Granular activated carbon (GAC) is also used as anode electrode due to its biocompatibility and low cost [244], [262], [263], [264]. The material is very porous and consequently the electrical conductivity remains quite low. Due to these characteristics, GAC is used mainly as a packing material rather than stand-alone anode. In order to increase conductivity, GAC has to be packed and this might lead to possible clogging in a flow-through MFC configuration. The overall surface area is quite high but the surface area available for bacteria interaction is rather low because the majority of the surface area available is in the nanometric scale. Usually, GAC is combined with carbon rods as current collector [244], [262]. Due to its intrinsic properties, GAC has very high surface area that can help the adsorption of organics pollutants or heavy metals. This property can be used for further purifying wastewater or trap heavy metals.

Granular graphite properties are similar to the GAC characteristics with the exception of a much lower surface area due to the lack of activation. Granular graphite has consequently a much higher electrical conductivity [265], [266]. As is the case for GAC, granular graphite is also used as packing material rather than stand-alone anode.

Carbonized cardboard is also a very interesting 3-D material composed by a single wall corrugated cardboard from recycled paper made from flute layer inserted between two liner layers and subject to thermal treatment (1000 °C) for 1 h in inert atmosphere. The obtained carbonized carboard is then attached onto a rigid support. The material is very low-cost, has high electrical conductivity and high porosity [191], [228], [267].

Graphite plate (or sheet) is a very simple electrode that guarantees high electrical conductivity and relative low cost. Graphite plate has low surface area and surface/volume ratio, and consequently results in lower output levels than porous or structured materials [268], [269]. It is often used as support for modified structures due to its high mechanical strength.

Reticulated vitreous carbon possesses unique characteristics being very conductive and have great porosity allowing the biofilm to penetrate through the entire structure and colonize the entire electrode. Unfortunately the material is quite fragile and very expensive to be used in MFCs [270], [271].

Other carbonaceous materials like electrospun carbon fibers [215], [272], [273], activated carbon nanofibers [274] and carbonized plant stems [275] have also been used as carbonaceous-based anode electrode.

As shown in Fig. 4, several metallic materials have been used as anode electrode in MFCs. Among them, stainless steel (plate, mesh, foam or scrubber) has been used with the main characteristic of being very conductive, robust and cheap [276], [277], [278], [279], [280], [281], [282]. More recently, other metals such as copper [229], [283], nickel [229], silver [229], gold [229] and titanium [230], [269] were also successfully investigated as anode electrode materials. Copper and nickel ions, released from electrodes, can be poisonous for microbes, and this has had negative effects on biofilm formation, yet high and stable performance levels have been reported [229].

Surface chemistry and surface morphology play a key role in the interaction between biofilm and anode electrode. A comprehensive review describes these phenomena [75] and summarizes the findings in Fig. 4 u (adapted). As shown in Fig. 4 u, there are three scales that can affect the interaction at the single-cell level, at the biofilm-level and at the integrated system-level [75]. Bacterial-level attachment can be improved by changing the surface chemistry such as i) surface charge [72], [284], [285] with positive charges usually preferred [72], [285]; ii) hydrophillicity/hydrophobicity with hydrophilic surface preferred during bacteria attachment [255], [286], [287], [288], iii) oxygen or nitrogen functional groups that facilitate bacteria/surface interaction [246], [289], [290], [291] and iv) immobilized mediators [75], [292], [293]. The attachment can also be affected by the surface morphology and the roughness that can be controlled at the nano-micro-scale [289], [294], [295]. Different chemical treatments [246], [296], [297], surface coatings [298], [299], electrochemical treatments [300], [301] and thermal treatments [227], [238], [302] have also been reported affecting both chemical and morphological surface area simultaneously or simply one of the two parameters. Modifications of electrodes have generally brought to positive effect with increase in the recorded output.

Moving to the biofilm-level, surface morphology plays a key role in the biofilm growth and the current produced. As can be seen in Fig. 4 u, the tendency is to move from a flat 2-D surface towards a 3-D electrode material in order to increase the available surface area and enhance the bio-interface between bacteria and electrode [75]. Theoretically, the increase of surface area should lead to a proportional increase in current. In a recent report, it was shown that the current produced by a carbon felt anode was higher than the current produced by a flat carbon graphite anode but there was no correlation between real surface area and current. This suggests that, among other reasons, not all the available area was successfully colonized by the biofilm or that full colonization, if possible, would need longer time frames [303]. Blanchet et al. [73] compared the biofilm formed on 2-D carbon cloth and 3-D carbon felt and the current produced. The results showed that despite the much higher surface area of the 3-D felt, the levels of performance were similar and the biofilm was not penetrating within the entire structure remaining on the outer surfaces [73]. The transition from a flat surface to a more complex 3-D surface implies also the possibility of facing limitations due to diffusion transport phenomena of both products and reactants and also relative pH gradients [304], [305], [306], [307]. These parameters seem to be of great importance for long term operation.

Finally at the system-level, the anode electrodes have to be designed in order to avoid clogging or “dead zone” [75]. This is mainly related with the design of the reactor and the overall system in order to guarantee long time operation. A more detailed discussion on this and other related parts can be found in Ref. [75].

### 2.5 Cathode catalysts and reaction mechanisms

The oxygen reduction reaction that is taking place at the cathode is often the limiting reaction of the MFCs and losses have been well identified and described in previous reviews [76], [123]. The oxygen reduction reaction (ORR) in neutral media can be facilitated by the utilization of enzymes [308], [309], [310], [311], [312], microbes [66], [313], [314] or abiotic catalysts [315], [316], [317], [318]. The latter seems to be the choice adopted in MFCs [315] and will be the focus of this review. These abiotic catalysts in particular, are mainly based on platinum-based materials, carbonaceous (metal-free) materials and platinum-group-metal-free (PGM-free) based materials on a carbon support [315], [316], [317], [318].

**Fig. 5:**
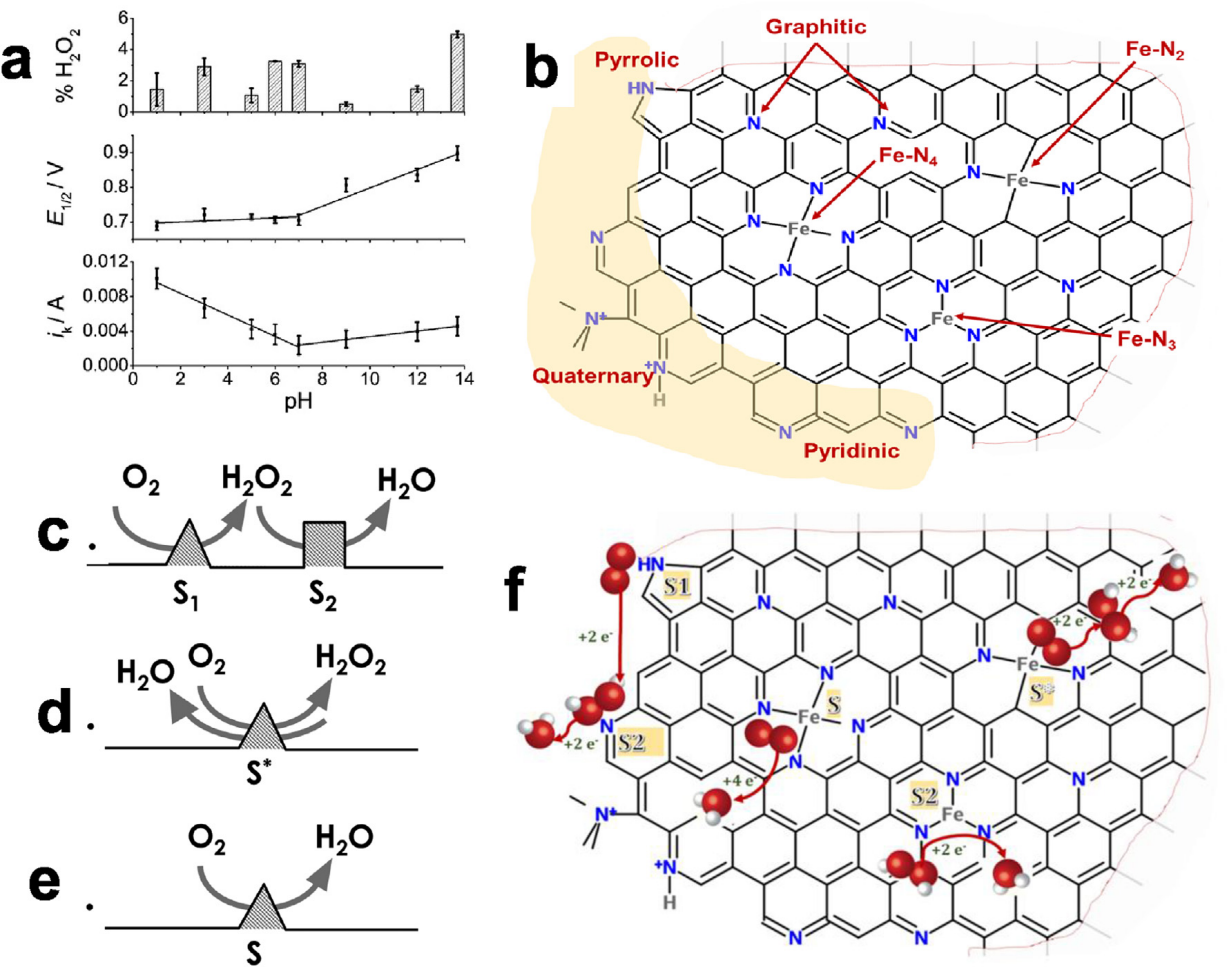
(a) H_2_O_2_ percentage (top); half-way potential for ORR (middle) and kinetic current ik (bottom) by Fe–4 phenantroline-based catalyst at a pH range of 1–13.7. (b) Schematic of N and Fe−Nx functionalities existing in Fe−N−C catalysts. Drawn of the possible reaction pathways and active sites during ORR: (c) 2x2e^−^ transfer on a dual site, (d) 2x2e^−^ transfer on a single site, and (e) direct 4e^−^ transfer mechanism on a single site. Identification of the active sites and the reaction pathways (f) with pyrrolic as S1 site, pyridinic as S2 site, and Fe-Nx as S or S* or S2 site. (Fig. 5a adapted from Ref. [322] with permission of Elsevier; Fig. 5b, c, d, e, f reprinted and adapted with permission from K. Artyushkova, A. Serov, S. Rojas-Carbonell, P. Atanassov, J. Phys. Chem. C 119 (2015) 25917−25928. Copyright (2015) American Chemical Society; Ref [339]).

The ORR involving abiotic catalysts can follow two different pathways (acidic and alkaline) that were previously well described in terms of reactions and relative redox potentials [219], [319], [320]. The acidic pathway implicates H^+^ and it has the intermediate production of H_2_O_2_ (involving 2e^−^) with the final product being H_2_O (involving 2 more electrons i.e. a total of 4e^−^ involved) [320], [321]. The alkaline pathway on the other hand, involves OH^−^ and it has the intermediate production of HO_2_^−^ + OH^−^ (involving 2e^−^) with the final product being OH^−^ (involving again 2 more electrons i.e. a total of 4e^−^ involved) [320], [321]. A 4e^−^ transfer mechanism is preferred since double the amount of electrons is transferred with the utilization of half of the reactant (oxygen) amount. In fact, in contrast to acidic or alkaline operating conditions, in which traditional chemical fuel cells work, the majority of BES work at circumneutral conditions that are actually the worst for ORR because of the lowest concentration of H^+^ and OH^−^ that are the main drivers for the ORR. A previous pH-dependence study based on Fe–4phenantroline that is a platinum-group-metal-free (PGM-free) catalyst, fabricated using a rotating ring disk electrode set up, showed the minimum kinetic current (i_k_) at pH 7 (Fig. 5a) [322]. It is not yet fully understood if the reduction reaction follows the acidic or alkaline pathway with final generation of H_2_O or OH^−^ respectively. Some reports have elucidated the presence of high concentration of OH^−^ in the cathode proximity [323], [324], [325].

In order to understand the electrochemical reaction mechanisms of the catalysts during ORR, rotating ring disk electrode (RRDE) technique is used [326]. A recent study in RRDE of a Fe-N-C catalyst showed that the acidic mechanisms shifted to alkaline at pH 11 [327] indicating that the acidic mechanism might be the one happening at the cathode. More detailed studies have to be done to better understand the process.

Interestingly, in the field of MFCs, the performance of catalysts incorporated into the cathode are better studied than the kinetics studies of the catalysts itself. To the best of the Authors' knowledge, only some studies have shown the electron reaction mechanisms pathway using RDE or RRDE in neutral media [328], [329], [330], [331], [332], [333], [334], [335], [336], [337], [338]. It is well know that Pt catalyst at neutral media facilitates a direct 4e-transfer mechanism with very low production of intermediate products measured by the ring current produced, as reported in the literature [328]. On the contrary, carbonaceous materials (without atomically dispersed metals) such as activated carbon or carbon black follow a 2e^−^ transfer mechanism [331], [335], [338]. Furthermore, the intermediate product was much higher (up to 50–70%) proved by high values of ring current measured. Interestingly, an increase in loading of the catalyst on the disk electrode, decreased substantially the peroxide formed with lower current produced at the ring electrode and this was explained with the trapping of the peroxide inside a thicker catalyst layer that was reduced before reaching the ring electrode [331]. Activated carbon has higher activity than carbon black, possibly due to a larger surface area, which in turn enhances the ORR kinetics [331], [335], [336], [338]. In other metal free catalysts, it was found that pyrrolic moieties enhance both the performance and the H_2_O_2_ production due to the 2e-transfer mechanism [339].

A more complicated, and debated topic is the electron transfer mechanism related with the PGM-free based materials and particularly the M-N-C catalyst with M being an earth abundant metal. In order to understand the electron mechanism, the chemistry of the materials has to be fully studied and understood. Several aspects of M-N-C catalysts like active sites structure, chemistry and geometry are still under discussion in the scientific community, but there seems to be consensus on the fact that the functionality of nitrogen and the specific metal on the surface, govern the oxygen reduction reaction. These functionalities related to M-N-C catalyst are well discussed in [339] and presented here in Fig. 5 b. From Fig. 5 b, it can be seen that graphitic nitrogen and Fe-N_x_ (X = 2, 3, 4) can possibly be formed as in-plane defects; in addition, pyridinic, pyrrolic, quaternary and Fe−N_2_/Fe−N_x_ can be considered possible edge sites of the catalyst [339]. Previous studies, which were based on theoretical calculations, concluded that nitrogen incorporated in carbon matrix enhances the electronic properties [340] but not the direct 4e^−^ ORR [341]. Artyushkova et al. studied a number of Fe-N-C catalysts and fewer metal-free (N-C) in acidic media identifying the dominance of the nitrogen and iron functionalities [339]. These findings are summarized in Fig. 5c and d. The conclusions were that: i) Pyrrolic N (S1) is responsible of a 2e^−^ transfer that reduces O_2_ to H_2_O_2_; ii) Pyridinic N (S2) is instead responsible for the further 2e^−^ reduction from H_2_O_2_ to H_2_O; iii) Fe-Nx can be responsible for: a) direct 4e^−^ reaction (S); b) 2x2e^−^ transfer mechanism on a single site (S*) and the 2e^−^ reduction from H_2_O_2_ to H_2_O [339].

**Fig. 6:**
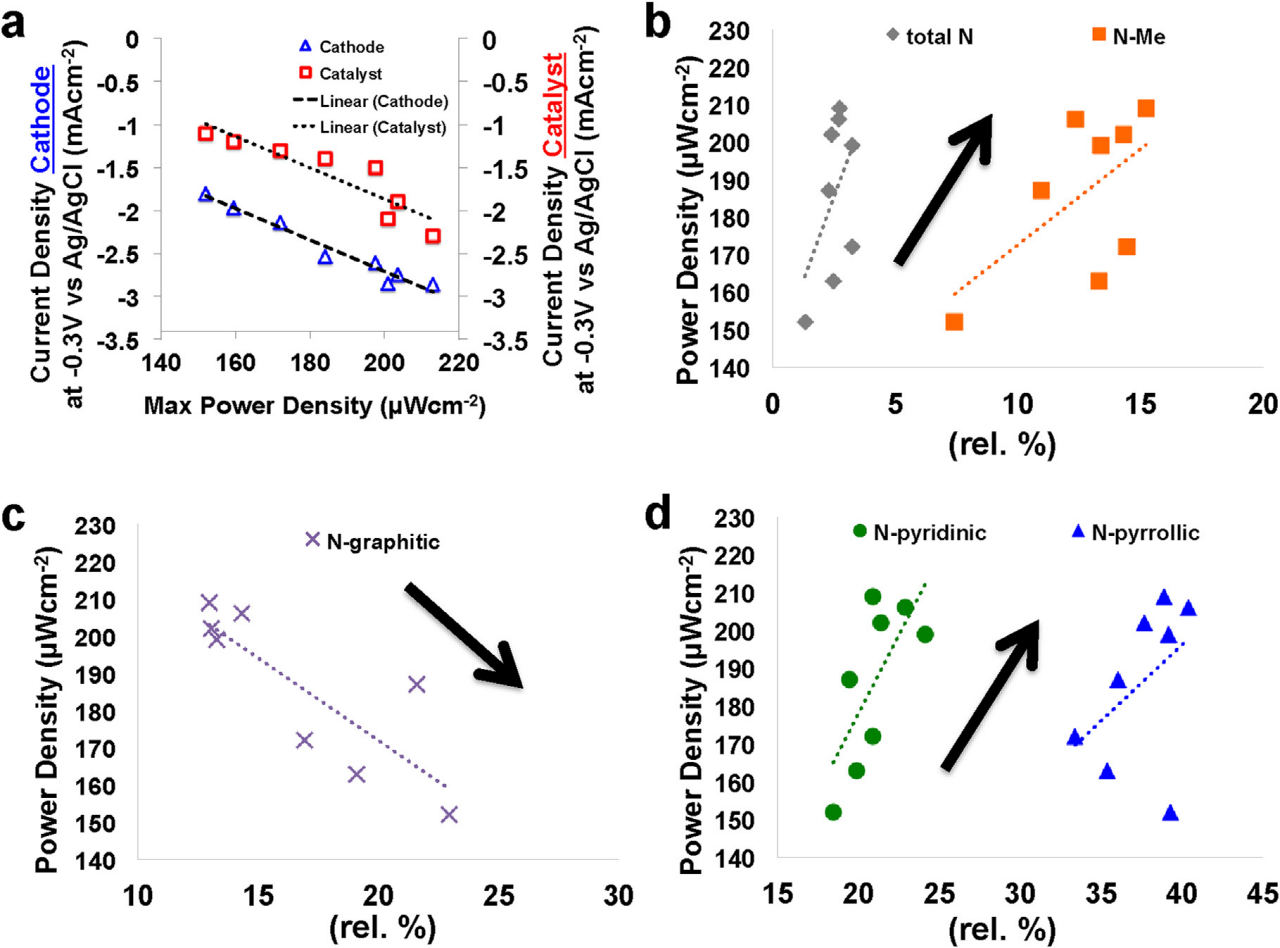
Relationship between the current from the RRDE and the air breathing cathode current with the maximum power density achieved by the MFCs using eight Fe-N-C catalyst (a). Maximum power density related with iron and nitrogen functionalities. Relationship with: (b) total nitrogen and nitrogen coordinated with the metal, (c) graphitic nitrogen and (d) pyridinic and pyrrolic nitrogen. (Fig. 6a, b, c, d rearranged and adapted from Ref. [329] with permission of Elsevier).

Several studies were conducted on RRDE using M-N-C catalysts in neutral media [328], [329], [330], [332], [333], [334], [337]. Among these, Fe-based catalysts were shown to have high activity and a 4e^−^ mechanism with relatively low H_2_O_2_ production. A recent study compared four M-N-C with M = Mn, Fe, Co and Ni and N-C as an amino-antipyrine (AAPyr) organic precursor in RRDE with a neutral pH electrolyte. The findings revealed that Fe had superior performance with an apparent 4e^−^ mechanism while a 2x2e^−^ can better describe the electron transfer mechanism of Mn-AAPyr, Co-AAPyr and Ni-AAPyr [328]. More detailed measurements using the kinetic current densities through the Koutecky-Levich analysis for different loadings showed also a 2x2e^−^ mechanism for Fe-AAPyr despite a very low peroxide production and high disk electrode currents achieved. Lastly, recent investigations have focused on relating the RRDE performance with the surface chemistry of eight Fe-N-C catalysts synthesized by different organic precursors (N-C) [329]. Results on RRDE showed a linear relationship between the performance and the total nitrogen content, and pyrrolic and pyridinic nitrogen and nitrogen coordinated to the metal [329]. The relationship between RRDE performance and graphitic nitrogen was on the other hand negative [329]. In contrast with the findings in acidic media, pyrrolic nitrogen had a positive effect on the disk current measured. Interestingly, the disk current produced during RRDE tests was positive related to the current produced by the catalysts incorporated into an air-breathing cathode and the maximum power generated by the MFC (Fig. 6a). This is a very important finding because the RRDE performance can predict the cathode performance and the overall MFC power output.

**Fig. 7:**
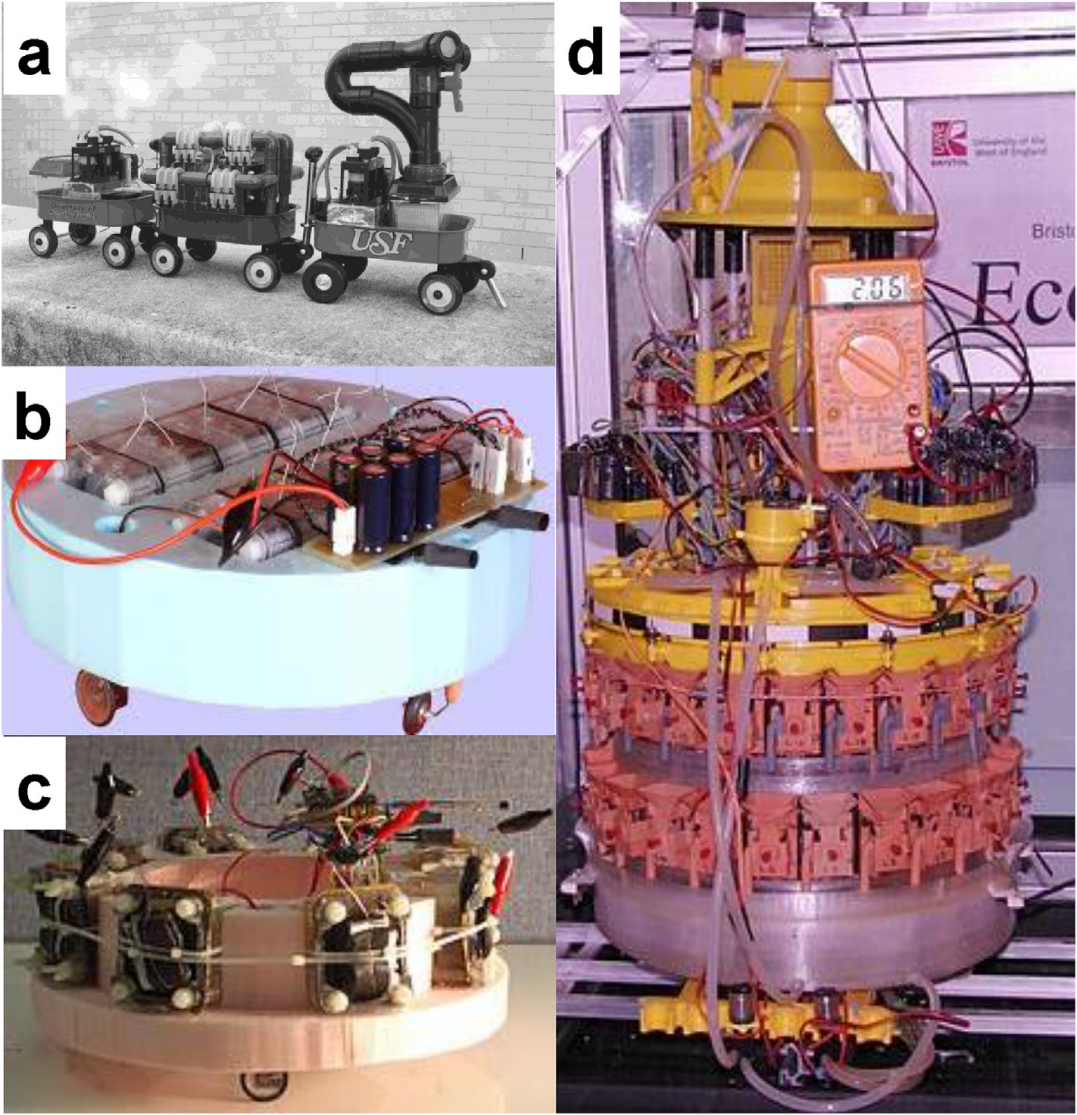
Digital photographs of Gastrobot, aka chew-chew train (University of S. Florida) (a), EcoBot-I (b), and EcoBot-II (c), each powered by 8 microbial fuel cells and EcoBot-III, powered by 48 small scale MFCs (d). (Fig. 7a Reprinted from S. Wilkinson, Autonomous Robots. 9 (2) (2000) 99–111 with permission of Springer, Fig. 7b, c and d source Wikipedia (https://en.wikipedia.org/wiki/EcoBot)).

Due to an increasing utilization of PGM-free catalysts working in neutral media and incorporated into MFC cathodes [315], future studies should perform more detailed investigations of the mechanisms involved and how catalyst kinetics may be improved.

### 2.6 Cathode materials and performances

Abiotic catalysts used in MFCs can be grouped in three main categories according to the function of the presence/absence of platinum and the presence/absence of earth abundant metals. These categories are: i) platinum-based (PGM-based) with a 4e^−^ transfer mechanism identified, carbonaceous-based (metal-free) with a 2e^−^ transfer mechanism identified and platinum-group-metal-free (PGM-free) with a more complex electron transfer mechanism (2e^−^ or 2x2e^−^ or 4e^−^) (Section 2.5).

These catalysts have been applied to different current collectors that can be carbonaceous based (e.g. carbon cloth, carbon paper, carbon felt, carbon veil, etc [227]) or metallic-based (e.g. stainless steel, titanium, nickel-chrome mesh [227]). Generally, the same materials described in Section 2.4 as anode materials can be also used as cathode current collector.

Several methods are used to apply or incorporate the catalyst into the cathode. These can be based on: i) spraying technique, ii) doctor blade technique, iii) drop casting, iv) pressing and v) rolling [342]. A review on air breathing cathode manufacture, materials (binders, diffusion layers, etc) and performances in MFCs was recently published [342]. The first three techniques are based on a preparation of a slurry or liquid solution that is applied on the current collector using a spray gun, a blade or directly through drops release respectively. Pressing and rolling techniques are instead based on the application of the catalyst on the current collector using pressure.

The first catalysts option is the utilization of platinum-based catalysts for ORR. Platinum based materials have been by far the most utilized for the cathode reaction [315] and now used often (and incorrectly) as controls compared to novel PGM-free or activated carbon based catalysts. Pt and Pt-alloy catalysts and cathode electrodes have been inherited by more mature technologies such as direct methanol fuel cells (DMFC) and hydrogen proton exchange membrane fuel cell (PEMFC) in which they are still considered to be efficient catalysts [343], [344], [345]. The cost is prohibitive and unsustainable for long term operations due to the low current/power produced, and durability is strongly compromised in a short time in the presence of anions and especially sulfur anions that are naturally present in wastewater [346]. Pt poisoning due to sulfur has been known since the 50′ [347]. Fast deactivation was measured due to the addition of sulfur [348], [349] and chlorine in the electrolyte media [350].

The second catalyst option based on carbonaceous materials seems to be viable for low cost practical applications, lowering the capital costs and having relatively high and stable performance [351]. Carbonaceous materials used as cathode catalysts are generally based on graphene [352], [353], [354], [355] (that is probably the most expensive among all the carbonaceous materials), activated carbon [356], [357], [358], [359], [360], [361], [362], [363], carbon nanotubes [364], [365], carbon nanofibers [366], [367], [368], [369], simple or modified carbon black [233], [370], [371]. It was shown that high surface area conductive carbonaceous materials and nanometric pores enhance the ORR in neutral media [320], [331]. Among the above-mentioned carbonaceous materials, activated carbon (AC) is by far the most used catalyst for ORR in MFCs [315]. Rolling [371], [372] and pressing [356], [357] techniques for preparing the cathode are the most common documented. The rolling method permits to create relatively larger cathodes but usually the pressure is not well controlled. The pressing method utilizing a professional press allowed a more controlled applied pressure despite lower cathode size can be fabricated [336], [373]. In both techniques, AC is previously mixed with a binder, generally polytetrafluoroethylene (PTFE), and then applied on the current collector [374], [375]. Several enhancements have been further pursued on optimization of AC based cathode. It has been showed that the different surface chemistry and porosity (meso and micro) affect significantly the performances [335], [376]. Moreover, also the temperature treatment at which the AC and PTFE are exposed affects significantly the performances [358], [359]. It has been showed that performances decreased when the temperature is raised to the PTFE sintering temperature (340 °C) [359]. The best temperature treatment for AC-cathodes was found to be between 150 and 200 °C that corresponds to the glass-transition state of the PTFE in which a better interaction between AC and polymer takes place [358]. A further enhancement in the performance using AC-cathodes was driven by the addition of a small relative percentage of carbon black in the mixture to enhance the poor conductivity of the AC that led to a remarkable increase in output [377]. Long term performances of over one year duration using AC-based cathodes have been recently showed with relatively stable output with a decrease within 20% demonstrating the robustness of the materials working in harsh environment in direct contact with wastewater [378], [379].

The third option is related with the utilization of catalysts based on PGM-free materials on carbon support and lately it is the one that is capturing the interest of the scientists all over the world. PGM-free catalysts are based on a transition metal associated with carbon and nitrogen and indicated with the acronym of M-N-C in which M is usually Mn, Fe, Co, Ni. Several successful examples of Mn-based [380], [381], [382], [383], Fe-based [348], [349], [384], [385], [386], [387], [388], [389], [390], [391], [392], Co-based [393], [394], [395], [396], [397], [398], [399], [400] and Ni-based [393], [401], [402] cathode catalysts have been shown in MFCs.

M-N-C catalysts are generally synthesized mixing metallic salt and organic precursors rich in nitrogen and carbon [403], [404], [405]. In some cases, silica is added as template and then removed using HF or KOH using the so called sacrificial support method (SSM) in order to control the catalyst morphology [406], [407]. Few interesting reviews have described the state of the art of PGM-free cathode catalysts in MFC systems [315], [316], [317], [318]. Those M-N-C PGM-free catalysts have been extensively studied in acidic [408], [409], [410] and alkaline media [411], [412]. In acidic media, high gap still has to be overcome with Pt-based catalysts in which the latter are the best performing and considered the state of the art. At the contrary, in alkaline media, PGM-free materials perform similarly or even better than Pt-based catalysts. The increase in interest for those materials is due to a substantial enhancement in performance with a slight increase in the initial capital cost [413]. PGM-free demonstrated high stability in heavily polluted environments outperforming PGM catalysts in MFC [348], [349], [414]. PGM-free are usually produced utilizing high temperature treatment (>900 °C) in which material pyrolysis take place [403], [404], [405], [415], [416], [417]. Fig. 5 b showed the nitrogen and iron functionalities of M-N-C catalysts synthesized using SSM. It has been showed recently that different organic precursors rich in nitrogen and carbon named guanosine, sulfadiazine, pyrazinamide, niclosamide, sulfacetamide, ricobendazole, quinine and succinylsulfathiazole used during the catalyst preparation affects significantly the catalytic performance and it can be strictly correlated to the surface chemistry of the latter [329]. Fig. 6b, c and d correlate the maximum power density with the nitrogen and iron functionalities [329]. At last, also the metal of the M-N-C catalyst influences the performances with Fe as the best PGM-free catalyst followed by Co, Ni and Mn [418].

In the last few years, M-N-C catalysts are often incorporated into AC-pellet air breathing cathodes in order to create an active and durable cathode for MFCs. Generally, PGM-free catalysts incorporated into AC-based cathodes showed outstanding performances between 1.5 and 2.5 Wm^−2^ [315], [316], [317], [318] with exceptions of cases in which the power was above 4.5 Wm^−2^ [334], [418]. These very high outputs can be justified by: i) positive and unnatural working conditions with high solution conductivity of the electrolyte, ii) high operating temperature and optimized MFC design; iii) data reported in function of the anode [187]. All those conditions affect significantly the MFC performances [419].

Few long terms performances are presented for PGM-free catalysts with losses of 15% within a month operation using Fe-ricobendazole and Fe-niclosamide catalysts [349] and ≈30% using Fe-EDTA for over 16 months [414]. Interestingly, performances were restored up to 90% after cleaning the cathode with blended HCl [414] indicating that inorganic and organic fouling plays an important role in diminishing the cathode activity over time [420].

### 2.7 Membrane materials

Membrane as well as structural materials, play a critical role in continuing the development of MFCs, especially with practical implementation in mind. This is because the architecture, choice of materials and overall geometry, significantly affect performance levels and unit cost. In this endeavor for better performance, with either readily available or bespoke materials, a number of investigations are ongoing in search of the optimum combination of “high performance/low cost/multi-functional” materials that can establish a formula for easy and economical scale-up. Initially, several examples concentrated on the utilization of cation-exchange-membranes (CEM) (e.g. Nafion) that was came from existing hydrogen PEMFC technology [421], [422] or membrane-based water treatment systems [270]. Due to the material's high cost, the majority of the work has then focused on finding alternatives that include trials with a range of materials such as j-cloth, nylon fibers, glass fibers, ceramics and biodegradable shopping bags [422], [423], [424], [425], [426], [427]. More unconventional materials, normally considered as waste such as natural rubber or laboratory gloves [428], have also been investigated [429], [430], and these materials appear to also offer advantages over membrane fouling [431]. Recently, it has also been demonstrated that a single finger piece from a laboratory glove, successfully operated a power management system, thus exhibiting practical implementation potential [249].

MFCs work on the principle of similar metals in dissimilar solutions, or dissimilar metals in identical solutions, since liquid electrolytes are used in the anode and cathode, provided that the cathode is not open to air. These liquid solutions contain ions, and so the ion-exchange-membrane is – in principle – not an essential requirement [432], provided that the anode and cathode are either dissimilar, which is the equivalent to electrochemical separation, or they are physically a certain distance apart, so that short-circuit can be avoided. Membrane-less MFCs have therefore already been described in the literature [433] and although this removes the need for expensive and prone-to-fouling membranes, it does suffer from oxygen diffusion and thus creating antagonistic competition with the anode for accepting the electrons.

The vast majority of MFCs consist of rigid, inert structural materials for housing the anode and cathode half-cells, regardless of whether these are membrane-based or membrane-less. It is more recently that rapid fabrication techniques, such as 3D printing, have been employed extensively for bespoke MFC architectures, designed for specific practical applications [434]. This has opened up the research into other more unconventional materials (as already mentioned above) and a positive outcome of this new – for MFCs – approach has been the testing of materials, which can have the dual purpose of being the structure as well as the ion-exchange-bridge. Rapid prototyping using 3D printing can also have the advantage of monolithic, complete MFC reactors, which widens the range of applications and environments that these can find use in Ref. [435]. In theory, any type of porous material, which has sufficient strength, chemical inertness and longevity could be employed as such, and this would also address the challenge of having the two electrodes close together, whilst avoiding oxygen penetration. Materials, such as microporous filtration membranes [436], canvas [437], nylon infused membrane [438] and photocopy paper [439] have been reported (a comparison of these types of material can be found in Kondaveeti et al. [440]).

One family of materials, which has shown great promise in this particular context of dual functionality, is ceramics. Terracotta, earthenware, mullite and so many other clay materials are ubiquitous in everyday life and in a variety of uses, dating from ca. 250 BC (or Parthian period). The same principles that apply to their development as products for the various markets (bathroom suites, tableware, garden pots etc.) for example, clay type and chemical composition, porosity - controlled by firing temperature, glazing for impermeability, thickness and shape/geometry, can also apply in MFCs, to give the ceramic material the features required for any specific application, including microbial colonization. A comparison of the different types of ceramic materials and how these have been used in MFCs, can be found in Winfield et al. [441].

### 2.8 MFC implementation in practical applications

Ever since the conception of the idea of a Microbial Fuel Cell by Michael C. Potter in 1910 [19], the technology has often had the reputation of a lab scientific curiosity, rather than that of a useful technology. This has had both a positive and a negative impact on the technology, which has nevertheless grown to become a scientific field in its own right (see Fig. 1). The positive impact has been the fact that numerous research groups are utilizing the MFC technology as a scientific tool for understanding microbial [442], biochemical [1], electrochemical [443] and material surface [246], [444] reactions under specific, controlled conditions, and investigate how these can be influenced by the choice of materials [1], feedstock substrates [15], [16] and chemical compounds [445], [446] among others. On the other hand, the negative impact has been the fact that the technology has never been considered as a serious contender in the wastewater treatment field [447] or in the renewable energy sector [447], even though it is perhaps the only example of a technology that can generate – rather than consume – energy from the cold oxidation of waste organic matter – and under certain conditions – inorganic carbon as well [448]. This means that compared to other technologies, MFCs have received less investment, in order to advance along the technology readiness level (TRL) scale. Despite this, there have been several reports of MFC implementation in various applications, precisely demonstrating the practical value of the technology. Several groups have studied MFCs as reactors for wastewater treatment with sizes growing from lab-scale (less than 1 L) up to thousands of liters [262], [449], [450], [451], [452], [453], [454], [455], [456], [457]. The first pilot scale (volume of 1 m^3^) with the purpose of treating brewery wastewater (Yatala, Queensland, Australia) was led by the Advanced Water Management Center at the University of Queensland and the images are shown in Ref. [43]. Interestingly, Ge et al. extracted energy from 100 L MFC reactor charging and discharging external ultracapacitors [449]. In these examples, the main goal was the detection of compounds or the degradation of organics rather than the energy recovery. In this review, the applications related with the energy harvesting and utilization are also described. The first example of microbial metabolism working in a real practical application is perhaps that of Gastrobot (aka Chew-Chew train) (Fig. 7a) [458]. This was a 3-wagon toy train, which employed an artificial stomach filled with *E. coli* (and HNQ as mediator) metabolising sugar, the reduced chemicals from which were fed into a stack of abiotic/chemical fuel cells, which produced energy to charge the bank of Ni-Cd batteries that were powering the train's motors and pumps.

**Fig. 8:**
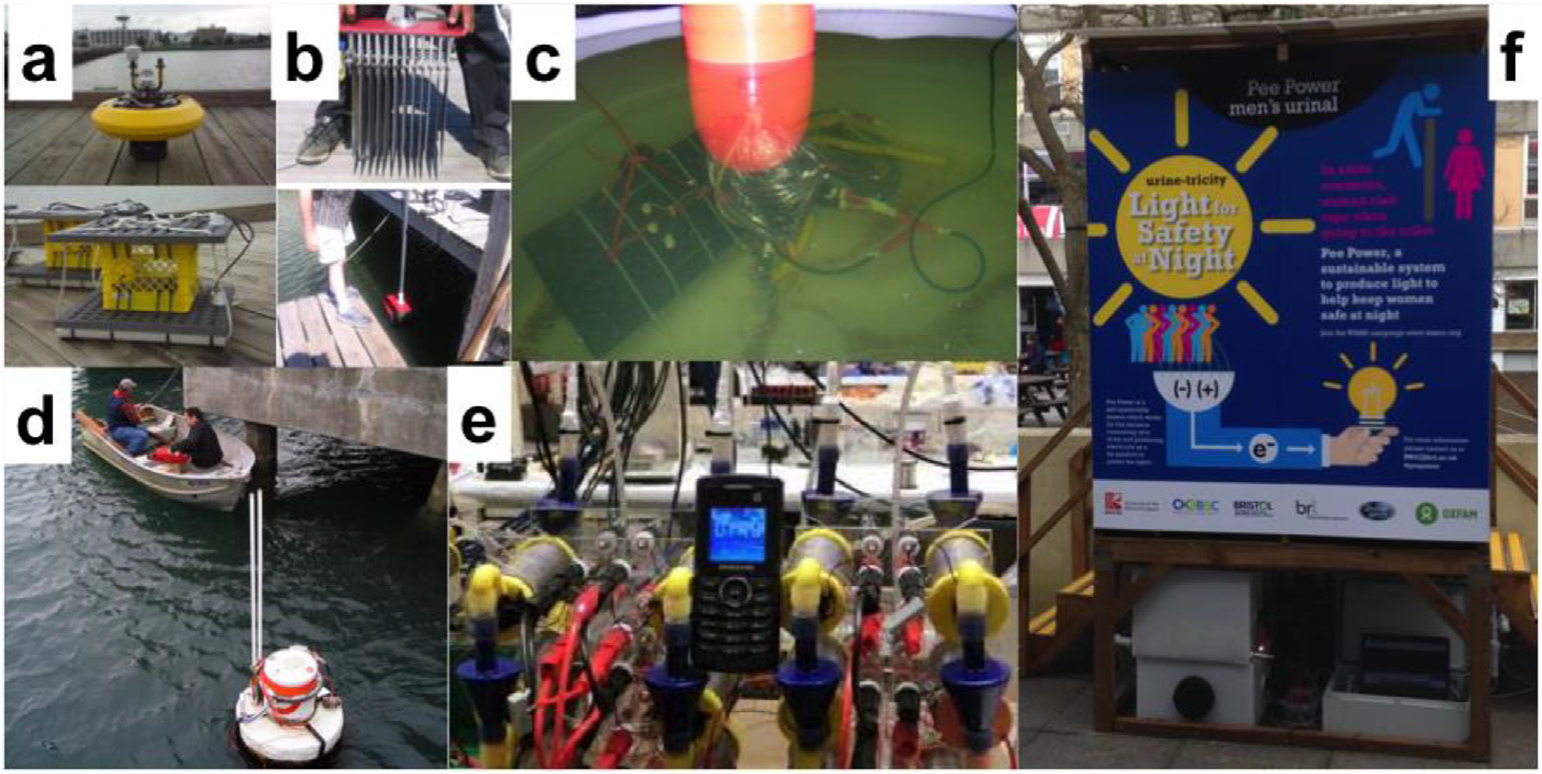
Images of the benthic microbial fuel cell done by Prof. Tender group (a,b) and by Prof. Beyenal group (c,d). A basic mobile phone charged by a stack of 12 ceramic microbial fuel cells (e), and the Pee Power™ urinal tested on the University of the West of England, Bristol campus (f). (Fig. 8a, b adapted from Ref. [461] with permission of Elsevier, Fig. 8c, d Photo Credit: Prof. Zbigniew Lewandowski and Prof. Haluk Beyenal, Fig. 8e Adapted from Ref. [473], published by the PCCP Owner Societies, CC BY 3.0 (https://creativecommons.org/licenses/by/3.0/)).

Two years later, EcoBot-I was reported as the first robot [225] which was directly powered by MFCs fed with glucose without using batteries, solar cells or any other form of conventional power source (Fig. 7b). EcoBot-I employed electrolytic capacitors for temporarily storing the energy from the MFCs aboard, and once full, the energy was released to actuate the locomotion motors and move towards the light (phototactic behavior). In 2005, EcoBot-II was reported [121], [459] as the next generation in the family of EcoBots, which in addition to being phototactic, was also able to report temperature wirelessly, without being dependent on chemical mediators (unlike EcoBot-I and Gastrobot) (Fig. 7c). These early proof-of-concept examples showed that it is possible to have artificial agents powered by microbial metabolism inside MFCs, but did not demonstrate the essential element of self-sustainability (or energy autonomy), since human intervention was still required to either replenish/replace chemicals and feed the MFCs. The first example of a self-sustainable robot that had its own circulatory system and was able to complete the thermodynamic cycle of ingestion-digestion-egestion was EcoBot-III (Fig. 7d) [434]. This robot demonstrated autonomy and simultaneously showed that miniaturizing the MFCs and multiplying the units into a stack, is one viable approach to scaling up the technology [460]. During this time, other examples of practical implementation have also been reported, in different environments. The first demonstration of MFCs doubling up as biological oxygen demand sensors for example, predates the Gastrobot/EcoBot development and this revealed the unique advantage of the technology to operate as a remote sensor. Benthic MFCs (Fig. 8a, b, c and d), for example, had also been subsequently reported for powering a meteorological buoy [461] (Fig. 8a and b), wireless temperature sensors (Fig. 8d) and other conventional environmental sensors [462], [463], [464], [465], [466]. Other types of microbial fuel cell have also shown the ability of powering environmental sensors [467], [468], [469], [470]. Several other applications have been reported including the first demonstration of a self-sustainable MFC stack [471], with sufficient energy to power its own feeding and hydration pumps and sustain long-term operation; the powering of a Texas Instruments Chronos digital wristwatch [472]; the charging of a basic mobile phone [473] (Fig. 8e) and more recently a smartphone [474] and LEDs for internal lighting [475]. This particular application has led to the Pee Power™ urinals [476] (Fig. 8f), which are being developed for trialing in refugee camps and slums, and is a line of work supported by Oxfam and funded by the Bill & Melinda Gates Foundation [476].

The drive for self-sustainability, and for demonstrating real work output in the true *Newtonian* sense, has pushed the technology envelope beyond what would have been otherwise done, to achieve the above-mentioned examples. Although these may still be considered as proof-of-concept exemplars, they are nevertheless showing that the MFC technology can have multiple applications, at multiple scales in multiple environments, by allowing the constituent microbes to do what they naturally do best: biotransform organic matter.

## 3 Perspective and directions

Microbial Fuel Cells have started as a scientific curiosity, and in many respects this remains to be the case. It is more recently that researchers have shown this to be truly a *platform technology*, which effectively means that MFCs can have multiple applications. To begin with, it is the only technology that can generate energy out of waste, without the input of external/additional energy, and this renders MFCs suitable for remote area access via the robotics route or remote power generation. Microbes are extremely sensitive, specific and accurate ‘sensors’ of their own environment, and the MFC is one of the very few technologies that can directly capture the microbial response and metabolism, and produce this as an analogue electrical signal. This gives the technology inherent sensing capability, which can be used in any environment, compatible with the microbes of choice. The use of ceramic materials as chassis and ion exchange membranes has been shown to produce or synthesise catholyte (liquid inside the cathode compartment), which has got disinfectant properties. The antagonism of the established biofilm on the anode electrode against any non-electroactive pathogenic organism results in the elimination of pathogens simply by exposure to the MFC environment. Both the latter two examples can improve sanitation, which is of particular interest for countries and regions of the Developing World. For many, and certainly from a funding/development perspective, MFCs are still a nascent technology but it is through continuous research into such technologies that we may find solutions to our global environmental problems, and MFCs will have a role to play in the future for the next generations and our planet. It is up to us to make it happen.
